# Engineering Metal-Organic Frameworks for Enhanced Antimicrobial Efficacy: Synthesis Methodologies, Mechanistic Perspectives, and Versatile Applications

**DOI:** 10.3390/jfb16090353

**Published:** 2025-09-19

**Authors:** Zaixiang Zheng, Junnan Cui, Shutong Wu, Zhimin Cao, Pan Cao

**Affiliations:** 1 School of Mechanical Engineering, Yangzhou University, Yangzhou 225009, China; 2 Institute of Intelligent Manufacturing and Smart Transportation, Suzhou City University, Suzhou 215104, China; 3 State Key Laboratory of Special Materials Surface Engineering, Wuhan Research Institute of Materials Protection, Wuhan 430030, China

**Keywords:** metal–organic frameworks, MOFs composition, bacterial resistance, AMR crisis, antibacterial efficacy, antibacterial mechanism, Synthesis Strategies

## Abstract

Bacterial contamination and the escalating crisis of antibiotic resistance represent pressing global public health threats, with approximately 4.95 million deaths linked to antimicrobial resistance (AMR) in 2019 and projections estimating up to 10 million annual fatalities by 2050. As third-generation antimicrobial materials, metal–organic frameworks (MOFs) have emerged as promising alternatives to conventional agents, leveraging their unique attributes such as high specific surface areas, tunable porosity, and controlled metal ion release kinetics. This review provides a systematic analysis of the foundational principles and core antibacterial mechanisms of MOFs, which include the sustained release of metal ions (e.g., Ag^+^, Cu^2+^, Zn^2+^), the generation of reactive oxygen species (ROS), and synergistic effects with encapsulated functional molecules. We highlight how these mechanisms underpin their efficacy across a range of applications. Rather than offering an exhaustive list of synthesis methods and metal compositions, this review focuses on clarifying structure–function relationships that enable MOF-based materials to outperform conventional antimicrobials. Their potential is particularly evident in several key areas: wound dressings and medical coatings that enhance tissue regeneration and prevent infections; targeted nanotherapeutics against drug-resistant bacteria; and functional coatings for food preservation and water disinfection. Despite existing challenges, including gaps in clinical translation, limited efficacy in complex multi-species infections, and incomplete mechanistic understanding, MOFs hold significant promise to revolutionize antimicrobial therapy. Through interdisciplinary optimization and advancements in translational research, MOFs are poised to drive a paradigm shift from “passive defense” to “active ecological regulation”, offering a critical solution to mitigate the global AMR crisis.

## 1. Introduction

Bacterial contamination constitutes a critical global challenge across food safety, healthcare, and environmental management sectors, contributing significantly to millions of annual fatalities and infections worldwide [1,2,3]. The escalating antimicrobial resistance (AMR) crisis, exacerbated by widespread antibiotic misuse [4], has been designated by the World Health Organization (WHO) as a paramount public health threat [5], with 2019 data implicating AMR in approximately 4.95 million global deaths—including 1.27 million directly attributed to drug-resistant infections—and disproportionately affecting low- and middle-income countries [6]. Projections indicate antibiotic resistance could cause 10 million annual deaths by 2050 [7], underscoring the urgent need for next-generation antimicrobial materials [8]. Consequently, antimicrobial agent development has progressed through three evolutionary stages: initial organic/inorganic compounds and salts [9], subsequent monometallic substances/metal oxides, and emerging engineered materials with programmable topological architectures [10]. Among these third-generation candidates, metal–organic frameworks have garnered significant attention [11], featuring exceptional specific surface areas [12], tunable pore geometries, and controllable ion release profiles [13]. Metal–organic frameworks are porous crystalline architectures constructed through the self-assembly of metal ions (or clusters) and organic ligands [13]. The diverse geometric configurations and bonding modalities of metal centers and ligands enable precise tailoring of their physicochemical attributes [14], allowing customization for specific applications [15], thereby underpinning the design of porous structures optimized for varied scenarios [16]. While early investigations into MOFs focused on gas catalysis, separation, and storage, their utility has expanded into realms such as energy storage, sensing, and biomedicine [17].

Synthetic strategies for MOFs have evolved in tandem with escalating application requirements [18], encompassing approaches such as solvothermal/hydrothermal synthesis [19], microwave-assisted fabrication [20], electrochemical methods [21], and mechanochemical techniques [22]. This diversity in synthesis, coupled with the flexibility of structural design, provides a robust platform for the development of next-generation antimicrobial materials [23]. Key advantages of MOFs in this context include: (1) serving as reservoirs for antimicrobial metal ions [24] (e.g., Ag^+^, Cu^2+^, Zn^2+^) with sustained release through framework degradation, ensuring prolonged activity; (2) high-efficiency encapsulation of antimicrobial agents [25] (e.g., tannic acid, curcumin, antibiotics) within their porous structures, enabling synergistic effects with metal ions [26]; (3) low toxicity, high biocompatibility, and modifiability, which address limitations of traditional metal oxide nanoparticles such as inherent toxicity and poor drug-loading capacity [27].

Despite the incomplete understanding of their antibacterial mechanisms, MOFs have made rapid strides in antimicrobial applications, including sustained antibiotic delivery, the development of antimicrobial coatings [28], and the design of composite nanomaterials. However, the evolutionary capacity of bacteria to develop resistance—via mechanisms such as efflux pump activation, enhanced antioxidant defense, and cell wall modification—poses a potential challenge to the long-term utility of MOF-based antimicrobials. This review synthesizes state-of-the-art research on MOFs and their composites in antimicrobial contexts, as shown in Table 1, encompassing: (1) prevalent MOF synthesis methods; (2) compositional features and antibacterial mechanisms of MOFs and MOF-based composites; (3) prospects and optimization pathways for MOF utilization in antimicrobial scenarios across medical, environmental, and food-related fields. It aims to inform the development of efficient, low-toxicity antimicrobial materials.

## 2. Synthesis Methods for MOFs

### 2.1. Hydrothermal Synthesis

**Figure 1:**
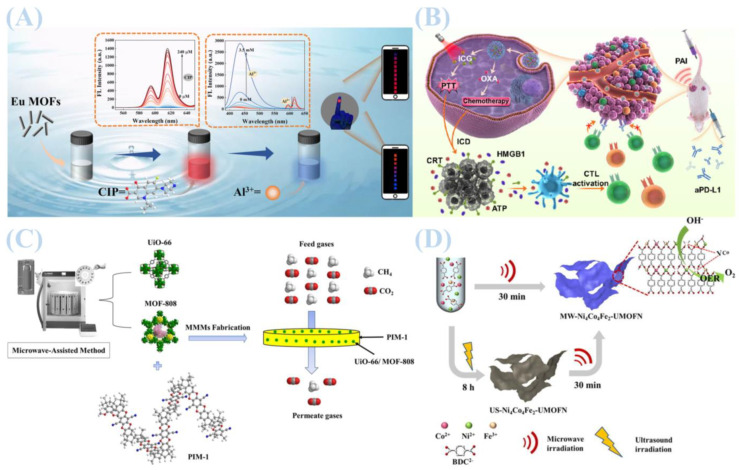
(**A**) Experimental mechanism diagram of neodymium metal–organic framework materials. Reprinted with permission from Ref. [31]. Copyright 2024 Elsevier. (**B**) MIL-100 nanoparticles were prepared using a microwave-assisted method and used as a drug delivery system for tumor treatment. Reprinted with permission from Ref. [37]. Copyright 2022 Elsevier. (**C**) Experimental mechanism diagram of metal–organic framework materials UiO-66 and MOF-808. Reprinted with permission from Ref. [35]. Copyright 2024 Elsevier. (**D**) Schematic diagram of the experimental principle for the preparation of ternary metal MOF nanosheets based on nickel-cobalt-iron using a microwave-assisted synthesis method. Reprinted with permission from Ref. [34]. Copyright 2021 Elsevier.

Hydrothermal synthesis is the most widely used method for fabricating metal–organic frameworks (MOFs) [29]. In this method, metal ions and organic ligands are combined in water or organic solvents at a specific stoichiometric ratio and heated in a sealed high-pressure vessel, such as an autoclave [30]. This process promotes the formation of MOFs with uniform morphology and high crystallinity. As illustrated in Figure 1A, Although it is a relatively straightforward approach that yields well-dispersed particles, its dependence on high temperature and high-pressure conditions presents challenges for scaling up production [31]. Additionally, the pore size and functionality of MOFs (e.g., MOF-808 (Hf)) can be tuned through ligand modification, enabling applications such as methane adsorption. These developments align with green chemistry principles and suggest a promising direction for future MOF synthesis.

### 2.2. Microwave-Assisted Synthesis

Compared with the conventional hydrothermal synthesis method, microwave-assisted preparation of metal–organic framework materials (MOFs) has been proven to be a more efficient synthesis strategy [32]. This method enables rapid and uniform heating of the reaction system, thereby significantly accelerating the reaction kinetics. However, localized overheating remains a critical challenge, as it may exert adverse effects on the nucleation and crystal growth processes of MOFs. To explore the intrinsic mechanisms underlying hotspot formation, recent studies have focused on simulating the temperature distribution under different synthesis conditions—including microwave input power and reaction chamber geometric configurations. By optimizing experimental parameters to alleviate the overheating phenomenon, researchers successfully increased the proportion of regularly shaped particles and narrowed the particle size distribution during the synthesis of MIL-88B(Fe) [33].

As illustrated in Figure 1D, Qiling Li and coworkers employed microwave-assisted synthesis to prepare nickel-cobalt-iron ternary metal MOF nanosheets. The resulting NiCoFe-MOFs exhibited exceptional oxygen evolution reaction (OER) activity in alkaline media, highlighting an effective strategy for modulating MOF electronic structures for electrocatalytic applications [34]. In another study, Zhenyu Zhao et al. replaced hydrofluoric acid with NaF as a modulator to synthesize MW-MIL-101(Cr) and MW-MIL-101(Cr)-NH_2_ [33]. This approach not only overcomes the limitations of traditional solvothermal methods—such as prolonged reaction times, reduced crystallinity, and low yields—but also outperforms other microwave-assisted strategies for MIL-family MOFs. It achieves superior crystallinity, phase selectivity, and productivity (0.5–0.7 g with >70% yield within 1 h; spatiotemporal yield > 1200 kg/m^3^/day). Notably, NaF-mediated regulation in microwave reactions enables the stable nucleation and growth of MOF structures that are unattainable via conventional methods.

As shown in Figure 1C, Mohamed Yahia et al. synthesized UiO-66 and MOF-808 using sustainable microwave-assisted technology, which were then incorporated as fillers into a PIM-1 matrix to fabricate mixed matrix membranes (MMMs) [35]. The results revealed that these MMMs exhibited significantly enhanced CO_2_ permeability and CO_2_/CH_4_ selectivity compared to pure PIM-1 membranes, with separation performance improving further at higher filler loadings. Additionally, microwave-assisted synthesis has been utilized to produce phase-pure ZIF-9 and ZIF-9@xGO composites for applications in hydrogen evolution reactions coupled with benzyl alcohol electrooxidation to benzoic acid. Studies indicated that in situ-generated Co(OH)_2_/CoOOH hybrids on the ZIF-9 surface act as catalytically active sites (with ZIF-9 serving as a precatalyst), while graphene oxide (GO) facilitates electron transfer. This catalyst system showed excellent performance across various substrates, with ZIF-9@10GO demonstrating superior activity due to its high specific surface area and abundant exposed active sites—findings that provide valuable guidance for designing high-efficiency electrocatalysts [36]. As depicted in Figure 1B, Liu et al. prepared MIL-100 nanoparticles via microwave-assisted synthesis and validated their utility as a drug delivery system for tumor therapy [37].

### 2.3. Chemical Synthesis

**Figure 2:**
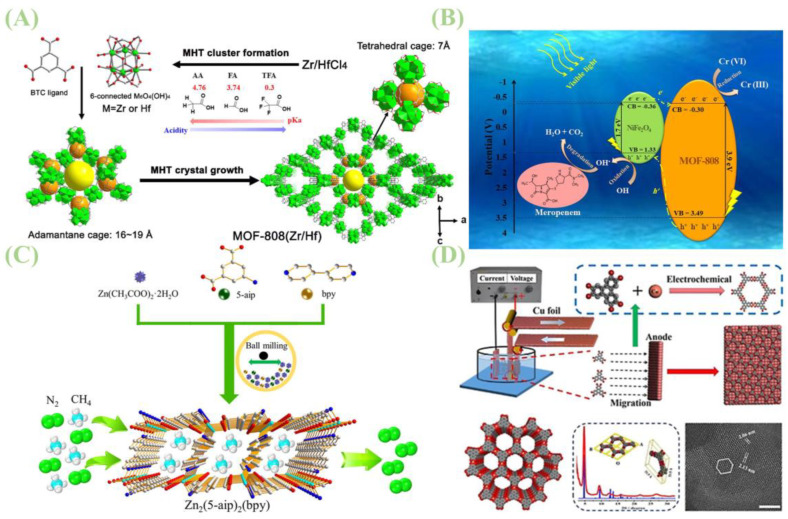
(**A**) Scheme of the MHT synthesis of MOF-808 with spn topology. Reprinted with permission from Ref. [39]. Copyright 2020 American Chemical Society (**B**) Experimental mechanism diagram of the synthesis of highly efficient NiFe_2_O_4_/MOF-808 hetero-junction photocatalysts using a mechanochemical method. Reprinted with permission from Ref. [47]. Copyright 2022 Elsevier (**C**) Mechanical chemical synthesis of zinc-based columnar metal–organic framework materials for efficient CH_4_/N_2_ separation. Reprinted with permission from Ref. [45]. Copyright 2019 Elsevier. (**D**) Experimental mechanism diagram of large-area Cu_3_(HHTP)_2_ MOF thin films prepared on single-crystal Cu(100) anodes using charge-induced molecular assembly. Reprinted from Ref. [43].

Chemical routes for MOF synthesis encompass electrochemical and mechanochemical approaches [38]. As illustrated in Figure 2A, Recent advances, such as modulated hydrothermal (MHT) synthesis, have improved the environmental friendliness and scalability of this method. For example, MHT has been used to produce MOF-808 at a high rate of 1.8 g/h with a remarkable space-time yield of 86.4 kg/m^3^·day [39]. Electrochemical synthesis modulates the release of metal ions via anodic oxidation or ligand reduction via cathodic reactions, enabling the directed assembly of MOFs in solution [40]. In contrast, mechanochemical synthesis employs mechanical forces from ball milling or grinding to drive direct reactions between metal salts/oxides and organic ligands, with little to no solvent required [41].

P. Arul and colleagues developed a strategy to fabricate 3D nucleated Cu-MOF (MPsLCu-MOF)-single-walled carbon nanotube (SWCNT) composites on glassy carbon electrodes (GCE) through electrodeposition. These composites were applied as sensors for sensitive glucose detection in human saliva, with the modified electrode achieving selective detection even in the presence of high concentrations of physiological compounds and metal ions [42]. As shown in Figure 2D, Youxing Liu et al. addressed the challenge of preparing large-area two-dimensional MOF films by proposing a novel electrochemical (EC) synthesis method. This approach leverages charge-induced molecular assembly to fabricate large-area Cu_3_(HHTP)_2_ MOF films on single-crystal Cu(100) anodes, which exhibit high crystalline quality and an electrical conductivity of ~0.087 S cm^−1^—approximately 1000-fold higher than that of the same material prepared via the interface method [43].

Mechanochemical reactions rely on solid reagents directly absorbing mechanical energy during grinding or milling. In typical ball milling, reaction energy is supplied by friction and impact between balls and reactants; high-energy grinding induces structural stress, bond cleavage, and formation of active free radicals, exposing reactive atomic layers at solid interfaces to accelerate reactions. Common techniques include solvent-free dry grinding (NG), liquid-assisted grinding (LAG), and ionic liquid-assisted grinding (ILAG). Negin Khosroshahi et al. synthesized an efficient NiFe_2_O_4_/MOF-808 heterojunction photocatalyst using simple mechanochemical techniques, which was applied to meropenem degradation and hexavalent chromium reduction under visible light, as shown in Table 2 [44]. The photocatalyst maintained high cycling stability over eight cycles, with no loss in catalytic efficiency after multiple recoveries. As shown in Figure 2C, Yongwei Chen et al. developed an efficient, eco-friendly solvent-free mechanochemical method to prepare zinc-based columnar MOFs in minutes, which was successfully applied to high-performance CH_4_/N_2_ separation [45]. Ana I. Martín-Perales et al. synthesized Ag- and Cu-modified ZIF-8 via continuous-flow mechanochemistry, realizing efficient continuous fabrication of MOF materials [46]. Antimicrobial tests against *Escherichia coli*, *Staphylococcus aureus*, and Candida albicans showed that optimized Ag-Cu@ZIF-8 achieved complete bacterial inhibition; notably, Ag-Cu@ZIF-8 with 5% Ag and 5% Cu exhibited antifungal activity with a minimum inhibitory concentration (MIC) of 0.1 mg mL^−1^ [46]. As depicted in Figure 2B, Sala and co-workers fabricated a copper-based primary catecholate MOF (PC-MOF) via mechanochemical (MC) liquid-assisted milling—a rapid and environmentally benign approach [47].

Electrochemical approaches offer strengths in precise control and device integration but are constrained by scalability. In contrast, mechanochemical methods stand out for their green efficiency and high stability, rendering them suitable for industrialization, though they face challenges in improving crystallinity. Future synergistic optimization of these methods (e.g., mechanochemical pre-synthesis coupled with electrochemical post-modification) is anticipated to pave the way for green MOF manufacturing.

While diverse MOF preparation techniques have advanced significantly, their industrial-scale production remains hindered by critical challenges related to scalability, cost-effectiveness, and reproducibility. The hydrothermal/solvent-thermal method—currently the most mature approach—relies heavily on high-pressure reactors and consumes substantial energy. These drawbacks not only restrict its ability to scale up but also drive up production costs; additionally, inconsistencies between batches undermine the reproducibility of large-volume manufacturing.

The microwave-assisted method offers notable improvements in reaction speed and yield, yet it faces a unique issue: the hot spot effect within the reaction chamber causes uneven temperature distribution. This irregularity impairs the uniformity of product morphology and crystal quality, while also demanding rigorous design standards and precise process control for industrial-scale reactors—adding both complexity and cost to equipment requirements.

Electrochemical methods excel in precise process control and device integration, but their batch-operated mode and subpar spatiotemporal yields limit large-scale throughput. Furthermore, the cost of electrode materials and the inherent complexity of electrochemical systems present additional hurdles for industrial applications.

In contrast, mechanochemical methods have emerged as a promising “green” alternative, thanks to their minimal or zero solvent usage, mild reaction conditions, high scalability potential, and low energy costs. However, they currently suffer from a critical limitation: the resulting products typically exhibit low crystallinity, which is also difficult to control accurately. This issue compromises the consistency of MOF material properties and, in turn, creates a barrier to the reproducibility of final products.

Looking ahead, optimizing production processes through the synergy of multiple techniques—such as combining mechanochemical pre-synthesis with electrochemical post-modification—will be a key strategy to overcome these challenges and enable the green, cost-effective fabrication of MOFs.

## 3. Compositions of MOFs

MOFs are widely utilized as carriers for antimicrobial agents, leveraging their unique compositional characteristics, high porosity, and large specific surface area [49]. This carrier role not only endows MOFs with exceptional antimicrobial performance but also grants them multiple advantages in antimicrobial applications.

### 3.1. Single-Metal–Organic Frameworks

Single-metal–organic frameworks represent an emerging class of porous materials that integrate inorganic and organic components, formed through coordination interactions between metal centers and organic ligands [50]. The strong bonding between metallic and organic segments allows for diverse combinations [51], resulting in a wide range of MOF variants tailored to specific applications—with particular potential in antibacterial fields such as biotherapeutics. Common MOFs are typically classified based on the valence states of their metal nodes or clusters: monovalent (e.g., Ag, Cu) [52], divalent (e.g., Zn^2+^, Cu^2+^, Co^2+^), trivalent (e.g., Fe^3+^, Cr^3+^), and tetravalent (e.g., Zr^4+^, Ti^4+^) [53]. While early synthetic endeavors primarily focused on Zn^2+^ and Cu^2+^, the expansion of biomedical applications has shifted attention to metals with intrinsic antibacterial properties (Ag, Co, Zn, Fe), as these elements enhance the antimicrobial activity of MOFs. Organic ligands in MOFs include carboxylic acid-based (e.g., dicarboxylic acids, phthalic acid derivatives), phosphonic acid-based, N-heterocyclic (imidazoles, pyridines), cyanide-based, and metal-containing types [54]. These ligands impart versatile functionalities to MOFs: those bearing antimicrobial groups (aldehydes, phenols) exert bactericidal effects through ligand release; photochemically active ligands (porphyrins) enable photodynamic therapy capabilities; and multifunctional ligands, after modification, improve MOFs’ dispersibility, biocompatibility, and targeting efficiency.

### 3.2. Bimetallic Metal–Organic Framework Materials

Bimetallic metal–organic frameworks surpass the performance constraints of single-metal MOFs through the synergistic integration of dual metal nodes (e.g., Zn/Cu, Fe/Zr) [55] and intermetallic cooperative effects. Heterometallic bonding further reinforces the structural stability of these frameworks, while their bimetallic active sites enable hierarchical responses to microenvironmental stimuli, facilitating precise, on-demand sterilization [56].

**Figure 3:**
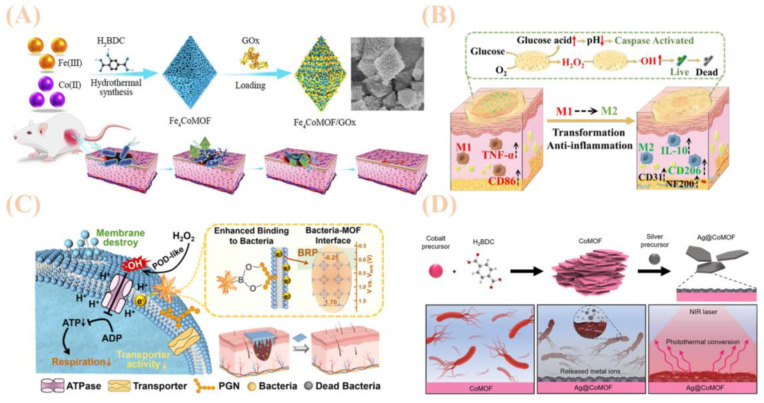
(**A**) Schematic diagram illustrating the mechanism by which the introduction of Co(II) into FexCoMOF enhances the electron transfer dynamics between Fe(III) and Fe(II), thereby significantly improving the activity of peroxidase (POD). Reprinted with permission from Ref. [60]. Copyright 2024 Elsevier. (**B**) MOF(Fe-Cu)/GOx-polyacrylamide hydrogel, mechanism diagram of the cascade catalytic system constructed by immobilizing glucose oxidase (GOx). Reprinted with permission from Ref. [59]. Copyright 2022 Elsevier. (**C**) Schematic diagram of t Its Application in Combating Bacterial Infection and Promoting Wound Healing. Reprinted with permission from Ref. [57]. Copyright 2025 American Chemical Society. (**D**) Schematic diagram of the mechanism of Ag@CoMOF-induced photothermal effect for sterilization through near-infrared (NIR) irradiation. Reprinted with permission from Ref. [56]. Copyright 2023 American Chemical Society.

A key advantage of bimetallic MOFs lies in their ability to enhance material performance via synergistic interactions between dual metal nodes, with design strategies focusing on structural modulation and functional enhancement. In exploring metal combinations and synergies, researchers select metal ions with complementary properties (e.g., Fe/Cu, Co/Zn, Mg/Cu) to optimize performance through intermetallic electron transfer, valence regulation, or cooperative catalytic effects. For example, incorporating Co(II) into FexCoMOF accelerates electron transfer kinetics between Fe(III) and Fe(II), significantly enhancing peroxidase (POD) activity, boosting hydroxyl radical (·OH) generation, and strengthening antibacterial potency [57]. Meanwhile, Ag@CoMOF triggers photothermal effects via near-infrared (NIR) irradiation (Figure 3D), enabling temperature-modulated sterilization and reducing the required dosage of antimicrobial agents [57]. Similarly, MgCu-MOF74 addresses the poor aqueous stability and weak antimicrobial activity of Mg-MOF74 via Cu^2+^ doping, while retaining the osteogenic activity of Mg^2+^ [58]. To expand functionality, bimetallic MOFs are often integrated with enzymes, nanoparticles, or polymeric materials (Figure 3C). For instance, Co/Zn-MOF@PDA-PAN electrospun membranes utilize polydopamine (PDA) modification to enhance MOF-substrate adhesion, improving material stability and prolonging antimicrobial longevity [58]. Additionally, some studies incorporate environment-responsive mechanisms to precisely regulate antimicrobial efficacy (Figure 3B): the Fe_4_CoMOF/GOx system, for example, responds to glucose concentrations to maintain optimal enzymatic activity under physiological pH, avoiding toxicity from exogenous H_2_O_2_ [59]. Despite significant progress in antibacterial applications, bimetallic MOFs face critical challenges. Long-term release of certain metal ions (e.g., Ag^+^, Co^2+^) may induce cytotoxicity, necessitating improved biocompatibility through ligand optimization or surface modification.

In complex biological fluids or environmental matrices [59], MOFs are prone to degradation due to fluctuations in pH and ionic strength, requiring enhanced structural stability designs such as hybrid composites or covalent cross-linking, as shown in Table 3. As illustrated in Figure 3A, furthermore, the molecular mechanisms underlying bimetallic synergies and in situ monitoring of reactive oxygen species (ROS) generation remain inadequately understood [60], highlighting the need for deeper investigation using in situ characterization techniques such as cryo-electron microscopy and Raman spectroscopy.

### 3.3. Multimetallic Metal–Organic Framework Materials

In recent years, multimetallic nanomaterials and metal–organic frameworks have garnered significant attention due to their unique structural tunability [61], component synergism, and superior physicochemical properties. These materials hold substantial application potential in fields such as environmental remediation, biomedicine, and energy conversion [62].

**Figure 4:**
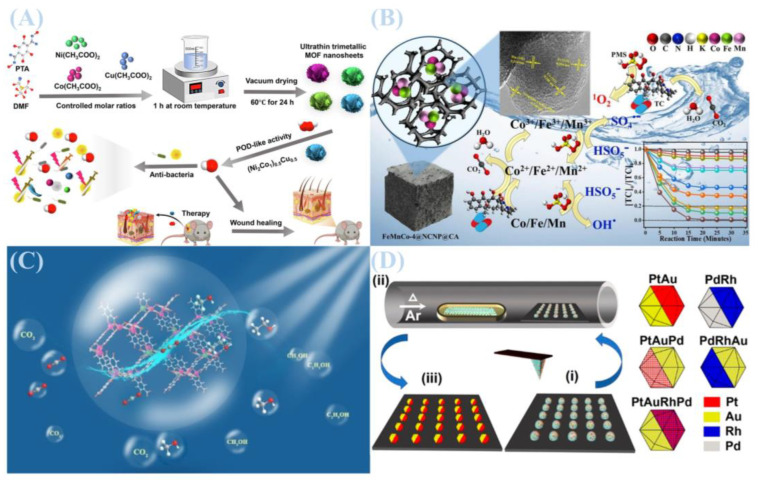
(**A**) Experimental flowchart for engineered ternary MOF nanosheets (NiCoCu-based MOF). Reprinted with permission from Ref. [64]. Copyright 2022 Elsevier. (**B**) Experimental mechanism diagram of the integration of tertiary metal-doped carbon nanoparticles based on MOFs with cellulose aerogels to activate peroxymonosulfate. Reprinted with permission from Ref. [68]. Copyright 2025 Elsevier. (**C**) Schematic diagram of the experimental principle of ultra-thin two-dimensional ternary metal MOF nanosheets (NiZrCu-BDC). Reprinted with permission from Ref. [69]. Copyright 2022 American Chemical Society. (**D**) Alloying/dealloying methods for preparing four-metal heterostructure nanoparticles (Pt, Pd, Rh, Au) with high-index crystal planes. Reprinted with permission from Ref. [70]. Copyright 2020 American Chemical Society.

In biomedicine, to address health risks posed by bacterial infections, researchers have developed diverse multimetallic MOFs and their derivatives [63]. For example, as shown in Figure 4A, tri-metallic MOF nanosheets (NiCoCu-based) leverage their peroxidase-mimetic activity to catalyze the production of toxic hydroxyl radicals (·OH), enabling efficient inhibition of Gram-positive bacteria [64]. These nanosheets also exhibit excellent biocompatibility in animal wound healing models. Additionally, based on ternary metal-deposited porous coordination network hybrid nanozymes [65], a colorimetric-fluorescence dual-mode detection platform has been established, allowing simultaneous specific recognition and elimination of bacteria, providing novel insights for managing environmental residual bacteria.

In food preservation, nickel/cobalt/iron ternary metal MOFs utilize their exceptional Fenton-like catalytic properties to construct high-efficiency antibacterial systems [66]. The composite coating formed with sodium alginate strongly inhibits Escherichia coli colonization on fruit surfaces, delays fruit weight loss, and thus offers a new strategy for developing food antibacterial agents [67]. In environmental remediation (Figure 4B), MOF-derived ternary metal nitrogen-doped carbon nanoparticles integrated with cellulose aerogels can activate peroxymonosulfate [68]. By harnessing synergies among multiple reactive oxygen species (O_2_, O_2_·^−^), they achieve 99.3% tetracycline removal efficiency within 15 min. These materials also demonstrate good reusability and environmental compatibility, presenting a sustainable solution for antibiotic wastewater treatment.

In energy conversion (Figure 4C), ultra-thin two-dimensional ternary metal MOF nanosheets (NiZrCu-BDC) enhance the efficiency of photocatalytic CO_2_ reduction to methanol and ethanol. This is achieved by enriching surface charges through metal doping and shortening electron transfer pathways. Their turnover frequency significantly exceeds that of bimetallic systems, highlighting the potential of multimetallic MOFs in carbon cycling and energy conversion [69]. Furthermore, progress has been made in the precise synthesis of multimetallic heterostructures (Figure 4D). Through alloying/dealloying approaches, four-metal heterostructure nanoparticles with high-index crystal faces (Pt, Pd, Rh, Au) have been prepared [70]. Combined with lithography technology, precise control over composition and size is realized, laying a foundation for designing high-efficiency catalysts.

These studies emphasize the critical role of multimetallic synergies in enhancing material catalytic activity, selectivity, and functional integration [71], providing strong support for interdisciplinary technological innovation and practical applications.

## 4. Antimicrobial Mechanisms of Metal–Organic Framework Materials

Bacteria are classified into Gram-positive (e.g., *Staphylococcus aureus*) and Gram-negative (e.g., *Escherichia coli*) strains based on cell wall structure: the former possess a thick peptidoglycan layer in their cell walls [72], while the latter have a thin peptidoglycan layer accompanied by an outer membrane composed of lipopolysaccharides. This structural difference results in varying tolerance to metal–organic frameworks: Gram-positive bacteria exhibit higher resistance, whereas Gram-negative strains are more susceptible.

**Figure 5:**
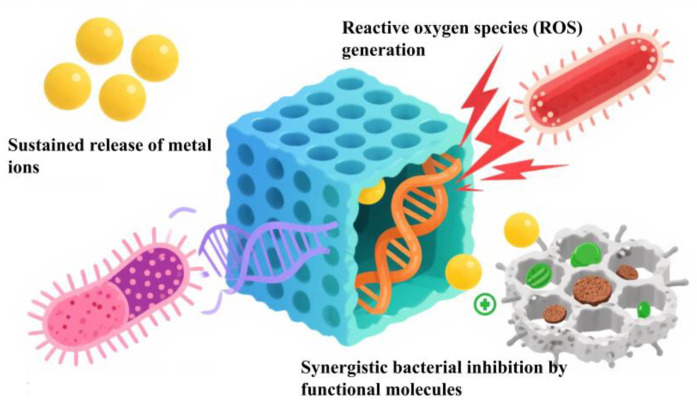
Schematic representation of various antibacterial mechanisms in metal–organic framework materials (MOFs).

Such discrepancies stem from two key factors: first, the thinner peptidoglycan layer in Gram-negative bacteria allows easier penetration of MOF-released ions into the cell interior; second, their negatively charged lipopolysaccharide outer membrane has a strong affinity for the predominantly positively charged ions released by MOFs. This interaction accelerates ion accumulation and induces intracellular damage [73]. The antimicrobial mechanism is shown in Figure 5.

### 4.1. Sustained Release of Metal Ions

MOFs exert antibacterial effects through a unique mechanism: their framework structure undergoes gradual degradation [74], enabling the sustained release of antimicrobial metal ions such as Ag^+^, Cu^2+^, Zn^2+^, and Co^2+^. These released ions act on bacteria through multiple pathways: they first disrupt the integrity of bacterial cell membranes, then interfere with intracellular processes like protein synthesis and nucleic acid metabolism, ultimately inhibiting or killing bacterial cells directly. Notably, the antibacterial efficacy of MOF-derived metal ions often surpasses that of traditional agents. For example, Ag^+^ and Co^2+^ released from Ag@CoMOF exhibit significantly stronger inhibitory effects against Escherichia coli and Bacillus subtilis compared to conventional antibiotics. Another instance is MgCu-MOF74, whose released Cu^2+^ responds to the acidic microenvironment generated by bacteria, enhancing its targeted antimicrobial efficacy [75].

In composite materials, MOFs retain their antibacterial functionality through metal ion release. For instance, HKUST-1/chitosan/PVA fibers achieve antimicrobial activity via the release of Cu^2+^ [76], while Ag@MOF/chitosan nanoparticles leverage the broad-spectrum antibacterial properties of Ag^+^. In the dual-layer composite dressing (Ag@MOF/CSNPs), the antibacterial core of the upper layer is the silver-based MOF (Ag@MOF) [77], which functions through Ag^+^ release. The mechanism of Ag^+^ action involves binding to bacterial cell membrane proteins, causing protein denaturation and inactivation, thereby disrupting membrane permeability. Additionally, once inside bacterial cells, Ag^+^ can bind to DNA, inhibiting its replication and thus suppressing bacterial proliferation [78].

However, it is important to note that the correlation between ion release rate and antibacterial efficacy is not always straightforward. Some studies suggest that excessive or overly rapid release may lead to cytotoxicity toward host cells, while others indicate that very slow release may fail to achieve effective bactericidal concentrations. Furthermore, the quantitative relationship between released metal ions and actual antimicrobial outcomes remains inconsistently reported across different MOF systems and experimental conditions.

### 4.2. Reactive Oxygen Species (ROS) Generation

MOFs, particularly bimetallic derivatives, display robust antimicrobial efficacy through the generation of reactive oxygen species (ROS). This process is mediated by either enzyme-mimetic activities (e.g., peroxidase-like catalysis) or photocatalytic reactions, producing ROS such as hydroxyl radicals (·OH) and singlet oxygen (^1^O_2_). These reactive species cause oxidative damage to essential bacterial components, including cell membranes, proteins, and nucleic acids.

Structural modifications in MOFs can enhance ROS-mediated antimicrobial efficacy. For example, Co-doped Fe_x_CoMOF exhibits heightened redox activity, facilitating increased ·OH production. Iron-copper bimetallic MOFs achieve localized ROS generation by targeting bacterial membranes, thereby improving antimicrobial precision. Ternary metal configurations, such as Ni_1_Co_1_Fe_1_-MOFs, leverage superior Fenton-like catalytic properties to generate highly oxidizing species (·OH) in reaction systems. These ROS disrupt bacterial structures—including Escherichia coli cell membranes—damage genetic materials (DNA/RNA), and interfere with intracellular metabolism, collectively enabling efficient bacterial inhibition.

Notably, this catalytic reaction-based antimicrobial mechanism circumvents the antibiotic resistance issues commonly associated with traditional antibiotics and chemical disinfectants, offering a promising alternative in antimicrobial strategies.

Reactive oxygen species (ROS) exhibit a “double-edged sword” property in biomedical applications. On one hand, they act as the core mechanism underlying photodynamic therapy and chemo-kinetic therapy, enabling efficient elimination of pathogens and tumor cells with broad-spectrum effectiveness. On the other hand, ROS can inflict damage on healthy host cells, as well as lipids, proteins, and DNA within these cells. Whether triggered by exogenous or endogenous stimuli, ROS possess significant therapeutic potential; however, they also tend to indiscriminately attack healthy host cells—impairing lipids, proteins, and DNA. This not only exacerbates inflammatory responses and hinders tissue regeneration and wound healing but also induces off-target effects, leading to severe collateral damage. Therefore, leveraging this double-edged sword relies on the development of precise regulatory strategies. Through implementing spatiotemporal control, ensuring microenvironmental responsiveness, enabling targeted delivery, and adopting combination therapies, ROS generation can be strictly restricted to localized lesion sites. This approach achieves a balance between maximizing therapeutic efficacy and minimizing adverse side effects.

Nevertheless, the quantitative assessment of ROS generation and its direct correlation with bactericidal efficacy remain contentious. Some reports question the specificity and reliability of common ROS detection methods (e.g., fluorescent probes such as DCFH-DA), which may yield false positives due to autoxidation or interaction with other reactive species. Moreover, several studies have demonstrated that ROS-independent mechanisms—such as physical membrane disruption or ion release—may dominate the antibacterial action in certain MOFs, suggesting that the relative contribution of ROS is system-dependent and requires careful validation under physiologically relevant conditions.

### 4.3. Synergistic Bacterial Inhibition by Functional Molecules

Studies have shown that a single antimicrobial mechanism—such as metal ion release or photodynamic effects—struggles to penetrate the defensive barrier of bacterial biofilm matrices at low concentrations. MOFs act as efficient carriers for antimicrobial agents, including tannic acid (TA), curcumin, and medicinal plant extracts. Their antimicrobial performance is enhanced through synergistic interactions between released metal ions and loaded components. For instance, tannic acid (TA) can coordinate with zinc-based MOFs, where the antimicrobial activity of metal ions combines with the polyphenolic structure of TA to disrupt bacterial biofilms, achieving a combined inhibitory effect.

Nano-MOF composite hydrogels loaded with copper nanoparticles (Cu NPs) and curcumin exhibit a multi-component synergistic antimicrobial mechanism: Cu^2+^ released from MOFs first damages bacterial cell membranes and DNA; the loaded Cu NPs, which possess stronger antimicrobial efficacy than Cu^2+^ alone, generate free radicals via oxidative stress to further destroy bacterial structures; meanwhile, curcumin inhibits the release of bacterial inflammatory mediators, alleviating infection-induced inflammation and indirectly reinforcing antimicrobial efficacy. This integrated system thus achieves synergistic anti-inflammatory and antibacterial effects, reducing infection risks.

MOFs can also combine with enzymes (e.g., glucose oxidase, GOx) to form cascade reaction systems, amplifying antimicrobial effects through multi-step catalysis. In the Fe-Cu MOF/GOx system, for example, GOx decomposes glucose to produce H_2_O_2_, which is then converted into ROS by the peroxidase (POD)-like activity of the bimetallic MOF. This enables an efficient “glucose → H_2_O_2_ → ROS” conversion process while avoiding toxicity associated with exogenous H_2_O_2_. With their microporous and mesoporous architectures, MOFs can efficiently load various antimicrobial agents, such as antibiotics, photosensitizers, and photothermal molecules. They deliver and release these agents at bacterial infection sites through carrier-mediated transport, exerting direct antimicrobial effects. Moreover, MOFs can co-load multiple drugs to achieve combined therapeutic effects and enhance antibacterial efficacy. For example, Zr-MOFs utilize their porous structure to increase the loading capacity of sage (SO) and marigold (CO) extracts, while regulating their sustained release to maintain long-term antimicrobial activity.

The porous structure of MOFs also enables controlled release of active components (e.g., metal ions and plant extracts), prolonging their antimicrobial duration. Additionally, their good compatibility with polymer matrices (e.g., chitosan and sodium alginate) enhances the biocompatibility of composite materials, which indirectly ensures sustained antimicrobial performance—this has been demonstrated in systems such as dual-layer composite dressings and modified wool fabrics.

Furthermore, while the direct antibacterial mechanisms of MOFs—such as ion release, ROS generation, and functional molecule synergy—have been extensively studied, their interactions with host immune cells remain underexplored. Immune cells, including macrophages and neutrophils, play critical roles in the clearance of pathogens and the resolution of infections. MOFs may modulate immune responses either by enhancing antibacterial activity through immune activation (e.g., promoting macrophage phagocytosis or neutrophil extracellular trap formation) or by inadvertently triggering chronic inflammation and tissue damage due to persistent immune stimulation. Future studies should systematically evaluate the immunomodulatory effects of MOFs to fully understand their impact on the host immune environment and optimize their design for improved biocompatibility and therapeutic outcomes.

## 5. Antimicrobial Applications of MOFs

MOFs, characterized by their high surface area and unique pore structures, have emerged as highly promising candidates for antimicrobial applications and drug delivery systems. Their key applications span wound healing dressings, medical antimicrobial coatings, and therapies targeting infections caused by antimicrobial-resistant bacteria. MOF-based composites enhance cell proliferation, angiogenesis, and matrix synthesis, while serving as carriers for bioactive molecules to boost tissue regeneration. Frequently integrated with other materials into structures such as hydrogels, fibers, and scaffolds, these composites exhibit multidimensional antimicrobial activity. Additionally, MOF coatings show substantial potential for surface modification in medical implants and related fields. Medical-grade MOF coating techniques—including in situ growth and immersion coating-enhance the performance of modified implant surfaces in reducing biofilm adhesion and resisting infections.

Methicillin-resistant *Staphylococcus aureus* (MRSA), a prevalent antimicrobial-resistant pathogen first identified in the 1960s and spreading rapidly from the 1990s onward, transmits via direct or indirect contact, attacking multiple organs and tissues to cause severe infections. Its treatment is challenging due to multiple resistance mechanisms (e.g., target-site mutations) and its ability to colonize intracellular niches of both phagocytic and non-phagocytic cells. While intravenous vancomycin remains a standard treatment, infections often recur, and intracellular MRSA is increasingly resistant to this antibiotic. Thus, the development of MOF-based nanodrugs and MOF-containing nanocomposites holds promising application prospects.

However, the long-term metabolic fate and biodegradability of MOFs within the human body have not yet been rigorously analyzed, which represents a critical gap in their clinical translation. Understanding the clearance pathways, potential accumulation, and breakdown products of MOFs is essential for ensuring their biosafety in biomedical applications.

### 5.1. Wound Dressings and Medical Coatings

MOFs are nanomaterials formed by coordination interactions between metal ions and organic ligands [79], resulting in highly porous crystalline structures. The materials offer numerous advantages, including ultra-high surface area, abundant active sites, low framework density, excellent mechanical, chemical, and thermal stability, as well as high porosity, tunable uniform size, and ease of functionalization. Such properties enable MOFs to efficiently adsorb and encapsulate bioactive molecules, drugs, and growth factors, achieving controlled release of therapeutic agents. Additionally, they can promote cell proliferation, angiogenesis, and tissue regeneration, thereby facilitating wound healing.

Wang et al. prepared HKUST-1/CS/PVA fibers (chitosan/polyvinyl alcohol) loaded with HKUST-1, a copper-based metal–organic framework (MOF), by co-hybrid electrostatic spinning [79]. The resulting material exhibited good physicochemical properties, biocompatibility, and antimicrobial activity. Preliminary experiments demonstrated its potential to promote repair in a total skin defect model. However, its long-term in vivo safety and the feasibility of its large-scale production remain to be evaluated [79]. Meng Zhang et al. developed a bilayered composite dressing comprising silver-loaded MOF/chitosan nanoparticles on the upper layer (Ag@MOF/CSNPs) and a polyvinyl alcohol/sodium alginate/chitosan (PACS) matrix on the lower layer [77]. While this system accelerated wound healing and reduced the inflammatory response in a mouse model, the stability of the bilayer structure and the potential cytotoxicity of silver ions still need to be investigated systematically. Cheng et al. reported on a TEMPO-oxidized bacterial cellulose (TBC)-based hydrogel containing an NIR-responsive antimicrobial system, which was created by incorporating TA-modified ZIF-8 (TA@ZIF-8) and MXene [80]. While this material showed strong antimicrobial capacity under 808 nm light, sufficient data were lacking to support its photothermal conversion efficiency, biosafety, and applicability in actual infected wounds [80]. Ya-Ling Fan et al. proposed using nano-MOFs as carriers and dynamic cross-linking agents with aldolized sodium alginate to construct a self-healing hydrogel [81]. This was then loaded with copper nanoparticles and curcumin to achieve synergistic antibacterial and anti-inflammatory properties. While this approach is innovative in terms of material design, the stability of the dynamic cross-linking mechanism, potential nanoparticle release toxicity, and in vivo efficacy still require further exploration [81].

Notably, the biodegradation behavior of these metal–organic framework (MOF)-based dressings and their metabolic clearance mechanisms have not been adequately investigated. Despite their promising regenerative function, their breakdown products and long-term biosafety must be systematically evaluated before clinical application, and this part of the results is derived from in vivo animal models rather than clinical trials.

MOF-based dressings are evolving from a “passive coverage” paradigm to an “active repair” era, with the core breakthrough lying in the synergistic integration of materials, technologies, and mechanisms. This advancement is manifested through three key strategies: leveraging composite material systems to enhance functional integration, adopting intelligent design principles to achieve precise spatiotemporal regulation, and utilizing biomimetic engineering to optimize biocompatibility. Moving forward, research should prioritize addressing critical challenges such as long-term toxicity mitigation and scalable manufacturing, while deepening investigations into regenerative medicine mechanisms—including fibrosis modulation and vascular-nerve co-regeneration—to accelerate the clinical translation of MOF-based dressings in fields like chronic wound management and tissue engineering.

As porous materials, MOFs possess distinctive traits that enhance performance when applied as thin films for biomedical surface coatings. A key feature of these coatings is biocompatibility, ensuring no adverse effects (e.g., inflammation or toxicity) during interactions with living tissues. Surface hydrophobicity and hydrophilicity—differentiated by a 65° contact angle—play a notable role in bacterial-surface interactions, host-material relationships, and processes such as osteogenesis, osteoblast adhesion, and proliferation. Developing antimicrobial coatings requires balancing broad-spectrum antimicrobial efficacy, biofilm inhibition, and biocompatibility, with biomolecules aiding MOFs in forming robust coatings in aqueous environments [82].

**Figure 6:**
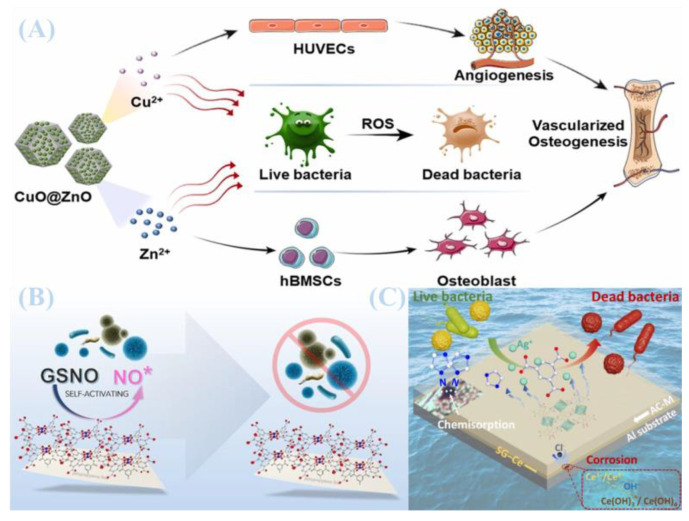
(**A**) Experimental mechanism diagram of CuO@ZnO composite materials grafted onto poly dopamine (PDA)-modified titanium alloy surfaces using metal–organic frameworks. Reprinted with permission from Ref. [86]. Copyright 2022 Elsevier. (**B**) Mechanism diagram of intelligent self-activated antibacterial coating, applicable to the preparation of medical-grade polypropylene (PP) surfaces. Reprinted with permission from Ref. [83]. Copyright 2023 Elsevier. (**C**) Schematic diagram of the mechanism of modifying silver-metal–organic framework (Ag-MOF) nanoparticles onto the acrylic coating of the AA2024 aluminum alloy substrate. Reprinted with permission from Ref. [87]. Copyright 2025 Elsevier.

**Figure 7:**
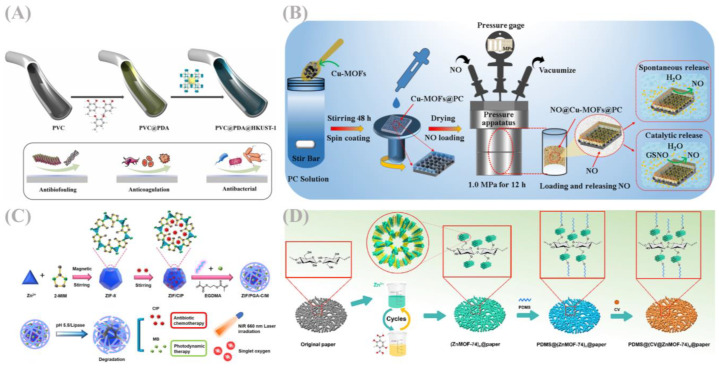
(**A**) Experimental mechanism diagram of anchoring Cu^2+^ containing coordination polymers (Cu-MOFs) on the inner surface of polyvinyl chloride (PVC) tubes Reprinted with permission from Ref. [84]. Copyright 2024 Elsevier. (**B**) Experimental mechanism diagram of spin coating to prepare amino-functionalized copper-based metal–organic frameworks (Cu-MOF) coatings on thermoplastic polyurethane (TPU) substrates Reprinted with permission from Ref. [85]. Copyright 2023 The Royal Society of Chemistry (**C**) Schematic diagram of the mechanism of chemotherapy-photodynamic combination therapy using novel metal–organic framework (MOF)/peptide hybrid nanocomposites for multidrug-resistant (MDR) infections Reprinted with permission from Ref. [88]. Copyright 2023 Elsevier. (**D**) Experimental mechanism diagram of metal–organic framework materials used as carriers for volatile antibacterial essential oils Reprinted with permission from Ref. [89]. Copyright 2023 Elsevier.

As shown in Figure 6B, Anastasia Skvortsova and colleagues developed a smart self-activating antibacterial coating for medical-grade polypropylene (PP) surfaces. Through covalent immobilization of a 3D periodic MOF (CuBTC), this coating enables efficient release of nitric oxide (NO) radicals from the endogenous donor S-nitrosoglutathione (GSNO), thereby conferring surface antifouling properties and suppressing bacterial adhesion to PP [83]. As shown in Figure 7A, Kangjia Sheng et al. utilized a bioinspired coating combined with layer-by-layer self-assembly to anchor Cu^2+^ containing coordination polymers (Cu-MOFs) onto the inner surface of PVC catheters [84]. The modified surfaces exhibited marked reduction in non-specific adsorption of model proteins (demonstrating superior anti-biofouling activity) and significant inhibition of platelet adhesion/activation (indicating excellent anti-platelet performance) [84].

As shown in Figure 7B, Zhang et al. fabricated amino-functionalized copper-based MOF (Cu-MOF) coatings on thermoplastic polyurethane (TPU) substrates via spin coating [85]. A polyurethane prepolymer coating (PC) enhanced interfacial adhesion between Cu-MOF particles and the TPU surface. Antimicrobial assays revealed that NO released from the coating exerted potent antibacterial effects against Escherichia coli and Staphylococcus epidermidis, with distinct inhibition zones and >96% antibacterial efficacy [85]. As shown in Figure 6A, Yunhui Si et al. grafted MOF-derived CuO@ZnO composites onto polydopamine (PDA)-modified titanium alloy surfaces [86]. The CuO@ZnO-coated titanium substrates effectively inhibited bacterial biofilm formation, achieving a 99% sterilization rate against Staphylococcus aureus. Leveraging the intrinsic porous architecture of MOFs, Zn^2+^ and Cu^2+^ were released in a controlled manner, promoting excessive intracellular reactive oxygen species (ROS) production in bacteria and ensuring exceptional antimicrobial efficacy of the composite coating [86].

Xie et al. synthesized Ag-MOF nanoparticles via a facile, mild liquid-phase route and integrated them into acrylic coatings on AA2024 aluminum alloy substrates (Figure 6C) [87]. These nanoparticles exhibited marked antimicrobial efficacy, attributed to the sustained release of Ag^+^ and their stable dispersion in the coating matrix, achieving population reduction rates of 95.9% and 87.2% against *Escherichia coli* and *Staphylococcus aureus*, respectively [87].

Despite these promising functionalities, the persistence and eventual metabolic processing of MOF coatings in biological environments—especially under chronic exposure—require further investigation. The potential release of metal ions or organic fragments over time must be carefully evaluated to preclude unintended cytotoxicity or immunogenic responses.

Engineering MOF-based antimicrobial coatings via pathogen-specific ligands (e.g., mannose and antibody fragments) enables targeted bactericidal activity, mitigates microbiota disruption, and integrates biosensing with feedback regulation to establish a “monitoring-sterilization-repair” closed-loop system. Integrating this platform with self-healing hydrogel technologies to prolong coating longevity remains a critical hurdle in meeting the lifelong antimicrobial demands of implantable devices. Moreover, understanding the degradation profile of such sophisticated coatings will be essential for ensuring their safety in long-term implantation.

### 5.2. Treatment of Drug-Resistant Bacterial Infections

MOFs possess considerable drug-loading capacities due to their microporous or mesoporous architectures, positioning them as nanocarriers for encapsulating diverse antimicrobial agents—including antibiotics, photosensitizers, and photothermal molecules—to achieve synergistic therapeutic effects. Alternatively, antibacterial moieties can be directly integrated into MOF frameworks as organic linkers. Furthermore, MOFs incorporate synergistic metal ions: coordinated cations such as Fe^2+^/^3+^, Cu^2+^, Zn^2+^, Co^2+^, and Ag^+^ within their structures, significantly enhancing intrinsic bactericidal cytotoxicity, yielding cooperative effects.

**Figure 8:**
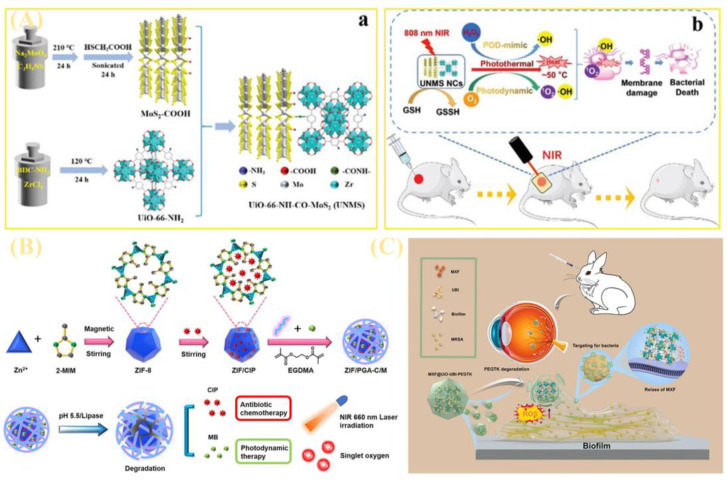
(**A**) Flowchart of the amidation reaction for the preparation of UiO-66-NH-CO-MoS_2_ nanocomposites (UNMS NCs) Reprinted from Ref. [91]. (**B**) Schematic diagram of the mechanism of chemotherapy-photodynamic combination therapy using novel metal–organic framework (MOF)/peptide hybrid nanocomposites for multidrug-resistant (MDR) infections Reprinted with permission from Ref. [92]. Copyright 2023 Elsevier. (**C**) Schematic diagram of the experimental principle of the novel antibiotic-loaded nanoparticle delivery system (MXF@UiO-UBI-PEGTK) Reprinted with permission from Ref. [88]. Copyright 2021 Taylor & Francis.

As shown in Figure 8C, Yu et al. engineered a novel antibiotic-loaded nanoparticle delivery system (MXF@UiO-UBI-PEGTK) comprising three key components: (i) a core of moxifloxacin (MXF)-loaded UiO-66 nanoparticles; (ii) the bacterial-targeting peptide ubiquitin (UBI29-41) immobilized on UiO-66; and (iii) a surface shell of ROS-responsive polyethylene glycol-thioether (PEG-TK) [90]. Subsequent evaluations characterized its key properties, including biocompatibility, toxicity, release kinetics, thermal stability, bacterial targeting capacity, and synergistic bactericidal efficacy against bacterial biofilms and endophthalmitis.

As illustrated in Figure 8A, Liao et al. fabricated UiO-66-NH-CO-MoS_2_ nanocomposites (UNMS NCs) via amidation. This positively charged material facilitates bacterial capture and confinement, with a distinctive feature: under 808 nm near-infrared irradiation, it integrates photothermal, photodynamic, and peroxidase-mimetic activities to exert synergistic effects [91]. Both its photodynamic properties and nanoenzymatic activity drive reactive oxygen species (ROS) generation, complemented by high catalytic activity across a broad pH range.

Liu and colleagues synthesized a novel gallium-based MOF at room temperature, followed by in situ loading of the model antimicrobial peptide melittin. The resulting nanocomposite exhibits stronger antimicrobial efficacy than pure melittin or gallium ions, achieving a “1 + 1 > 2” synergistic effect. It also shows favorable biocompatibility, accelerating the healing of methicillin-resistant Staphylococcus aureus (MRSA)-infected wounds by downregulating the inflammatory cytokines IL-6 and TNF-*α* [92].

**Figure 9:**
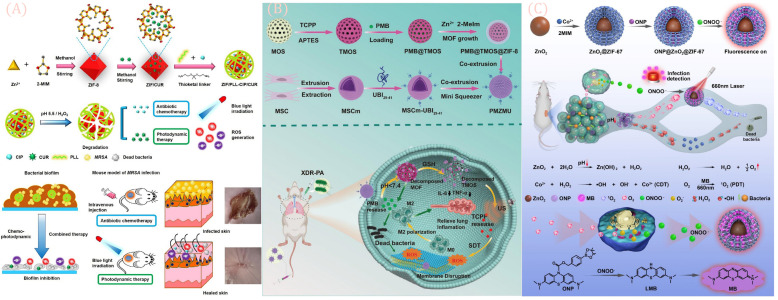
(**A**) Experimental mechanism diagram of active oxygen (ROS)-responsive drug release and photodynamic activity achieved by poly-L-lysine (PLL)-modified metal–organic framework (MOF) nanoparticles (ZIF/PLL-CIP/CUR) Reprinted with permission from Ref. [93]. Copyright 2024 Elsevier. (**B**) Engineering modification of human umbilical cord mesenchymal stem cells, experimental mechanism diagram of the preparation of nanomodified human umbilical cord mesenchymal stem cells. Reprinted from Ref. [95]. (**C**) Schematic diagram of the experimental mechanism of the novel ZIF-67-coated zinc oxide core–shell structure loaded with organic near-infrared probes (ONPs)Reprinted with permission from Ref. [97]. Copyright 2022 Elsevier.

As shown in Figure 9A, Xiang et al. developed poly-L-lysine (PLL)-modified MOF nanoparticles (ZIF/PLL-CIP/CUR), enabling ROS-responsive drug release and photodynamic activity [93]. PLL is linked to the zeolite imidazole framework (ZIF) shell via ROS-sensitive thione bonds to achieve controlled ciprofloxacin (CIP) release; curcumin (CUR), encapsulated within ZIF as a photosensitizer, generates singlet oxygen (^1^O_2_) and superoxide anion radicals (O_2_^−^) under blue light, effectively inhibiting MRSA. This nanoparticle combines chemotherapy and photodynamic therapy to eliminate MRSA and disrupt biofilms.

Given that multidrug-resistant (MDR) strains exacerbate infection-related mortality, morbidity, and treatment costs, Abdul Jabbar et al. prepared polydopamine-coated Zn-MOFs to enhance curcumin’s antimicrobial efficacy against *S. aureus* and *E. coli*. Smaller-sized MOFs efficiently load and release curcumin, and encapsulation within PDA-coated MOFs significantly boosts curcumin’s bactericidal potential—SEM confirms complete morphological distortion of bacteria post-treatment with PDA-Cur-Zn-MOFs [94].

As shown in Figure 7C, Xiang and co-workers also synthesized a novel MOF/polypeptide hybrid nanocomposite for chemo-photodynamic combined therapy of MDR infections. Using a lipase-sensitive crosslinker, they formed a crosslinked polypeptide shell within zeolite imidazole framework (ZIF)/poly-γ-glutamic acid (PGA) nanocomposites, creating an MRSA-targeted therapeutic system (ZIF/PGA-C/M). This composite potently inhibits both planktonic and biofilm-associated MRSA, with synergistic efficacy in a murine skin infection model [88].

As depicted in Figure 9B, Huang et al. engineered nano-modified human umbilical cord mesenchymal stem cells (hUC-MSCs)—termed PMZMU—as sonosensitizers for synergistic sonodynamic-nanoantimicrobial therapy against Gram-negative MDR bacteria [95]. PMZMU comprises: UBI29-41 (a bacterial-targeting peptide)-modified hUC-MSC membranes (MSCm), mesoporous organosilica nanoparticles doped with the sonosensitizer meso-tetra(4-carboxyphenyl)porphyrin, and acidity-responsive ZIF-8. This formulation efficiently loads polymyxin B, reduces off-target release, enhances circulation and targeting, and generates ROS under ultrasound. It reduces bacterial burden, mitigates inflammation by promoting M2 macrophage polarization, and improves survival without adverse effects. Chen et al. integrated bacterial-binding boronic acid ligands and photosensitive porphyrins into a single MOF, enhancing antimicrobial activity via synergism [96]. The introduction of boronic acid creates tight physical gaps, enabling the multivariate MOF to more effectively eliminate MDR bacteria. This work proposes a strategy for developing bacteria-targeted multivariate MOFs, inspiring future specific bacterial-binding therapies. As shown in Figure 9C, a novel ZIF-67-armored zinc peroxide core–shell structure was developed and loaded with an organic near-infrared probe (ONP)—ONP@ZnO_2_@ZIF-67 (ONP@ZZ). Acting as an “ROS factory,” this nanomaterial enables drug-resistant bacterial clearance via image-guided, in situ activated photodynamic therapy (PDT) [97].

Despite demonstrating exceptional antimicrobial properties in vitro and in animal models, the clinical translation of metal–organic frameworks (MOFs) remains in its nascent stages. Several research teams are currently advancing MOF-based materials into preclinical and early clinical trials, primarily focusing on topical formulations, implant coatings, and infection treatment systems. For instance, a ZIF-8-based dressing for diabetic foot ulcers has completed preliminary human safety assessments (NCT04855305), demonstrating favorable biocompatibility and significantly accelerated wound healing (with healing times reduced by approximately 30%). A Mg-MOF74 coating system for preventing orthopedic implant infections is currently undergoing Phase I clinical trials, with preliminary data indicating effective reduction in postoperative infection rates and enhanced osseointegration.

Furthermore, MOF drug delivery systems show promise in treating ocular infections (such as bacterial endophthalmitis) and periodontitis. For instance, the successful application of the moxifloxacin-loaded UiO-66 nanosystem in rabbit models provides a basis for its advancement into clinical studies. Nevertheless, the clinical implementation of MOF materials faces multiple challenges, including long-term in vivo toxicity assessment, large-scale standardized production, sterilization stability, and regulatory approval pathways. Future multi-center clinical trials are required to further validate their safety and efficacy, driving the substantive transition of MOFs from laboratory research to clinical application.

### 5.3. Other Antimicrobial Applications

**Figure 10:**
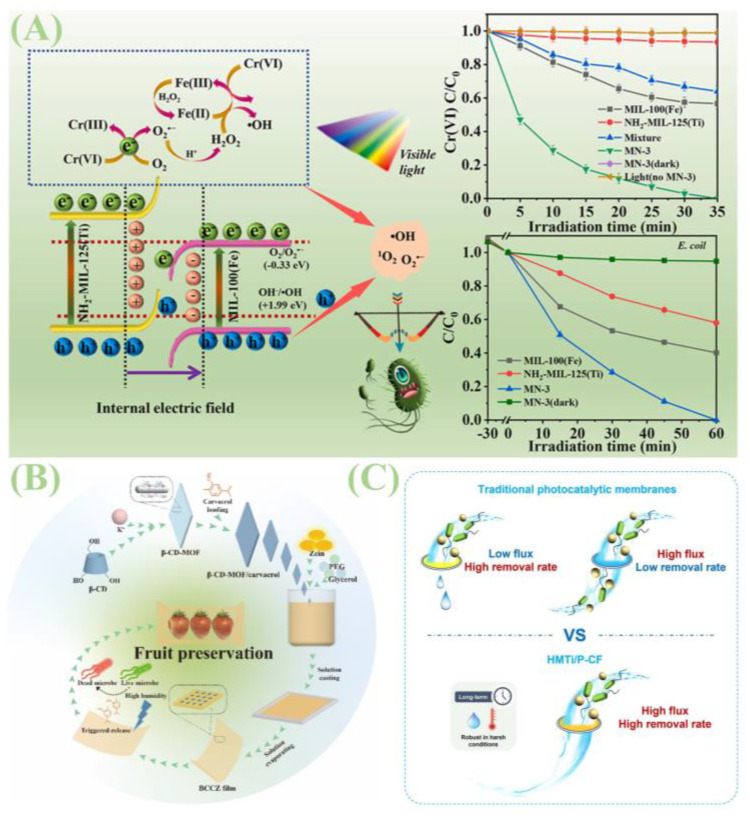
(**A**) Experimental mechanism diagram of visible light-driven Cr(VI) photoreduction and antimicrobial efficacy of novel MOF-on-MOF composite material (MN) prepared by ball milling processReprinted with permission from Ref. [99]. Copyright 2023 Elsevier. (**B**) Schematic diagram of the experimental principle of combining cellulose paper substrates with metal–organic frameworks Reprinted with permission from Ref. [98]. Copyright 2025 Elsevier. (**C**) Schematic diagram of the experimental principle of a photocatalytic membrane with high throughput and high removal efficiencyReprinted with permission from Ref. [100]. Copyright 2024 Elsevier.

MOFs have found extensive applications in the food industry, as shown in Table 4. Their superior antimicrobial properties offer novel solutions for addressing antibiotic resistance in foodborne pathogens. As shown in Figure 10B, Nong et al. engineered a BCCZ composite film by integrating carvacrol-loaded β-CD-MOF with zein (corn prolamin) [98]. Under high-humidity conditions, this film potently inhibits Gram-positive bacteria (e.g., *S. aureus*), Gram-negative bacteria (e.g., *E. coli*), and fungi (e.g., Penicillium). Yang et al. reported a facile, energy-efficient strategy that integrates cellulose paper substrates with MOFs to enhance hydrophobicity and confer long-acting antibacterial effects [89]. This work highlights the potential of in situ-formed MOF-doped coatings as a functional modification platform for fabricating active superhydrophobic paper-based packaging. Amid growing demands for food safety and quality, developing natural preservatives and their carriers has become a focus of novel preventive control strategies. As shown in Figure 7D, Yunpeng Wu and co-workers pioneered the use of MOFs as carriers for volatile antimicrobial essential oils, demonstrating that thymol-loaded Zn@MOF acts as an effective antimicrobial agent with potential indirect applications in the food sector [89].

Recent advancements have also been made in MOF-based materials for wastewater purification and disinfection. As shown in Figure 10A, Longyang Wang and colleagues prepared a novel MOF-on-MOF composite (MN) via simple ball milling [99]. This composite exhibits efficacy in visible-light-driven Cr(VI) photoreduction and antimicrobial efficacy, providing insights for constructing MOF-on-MOF heterojunctions to treat wastewater contaminated with Cr(VI) and bacteria. As depicted in Figure 10C, Hou et al. developed a high-flux, high-removal-efficiency photocatalytic membrane [100]. Specifically, they grafted dodecyl bis(2-hydroxyethyl)methyl ammonium chloride onto MIL-125(Ti)-NH_2_, then firmly immobilized the modified photocatalyst in a high-porosity nonwoven fabric via polyvinylidene fluoride phase inversion. Electrostatic capture enables pathogen interception independent of membrane pore size/porosity; additionally, perturbations from long alkyl chains and positive charges enhance photocatalytic inactivation. This membrane retains long-acting antimicrobial properties in darkness, inspiring efficient, large-scale, and sustainable water purification strategies.

**Figure 11:**
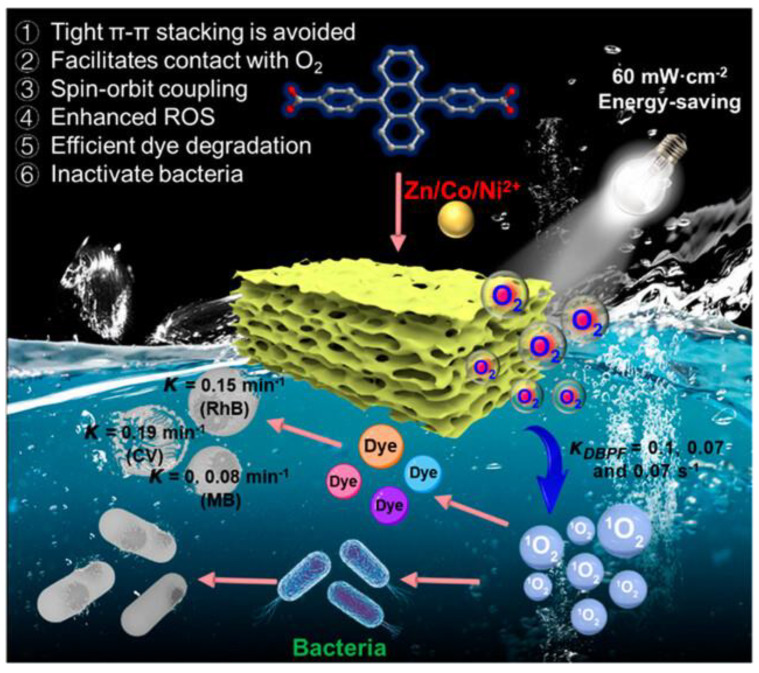
Experimental mechanism diagram of porous anthracene metal–organic framework materials (MOFs, namely GXNU-1, GXNU-2, and GXNU-3) used for water purification. Reprinted with permission from Ref. [103]. Copyright 2023 American Chemical Society.

Sha et al. synthesized carbon-MIL-125 via coupled hydrothermal and carbonization processes using pre-hydrolysate and TiO_2_ (Ti-MOF) [101]. This approach for fabricating carbon-based materials from pre-hydrolysate not only yields high-value-added products but also maximizes bioresource utilization, offering new prospects for designing high-efficiency water disinfection photocatalysts. Zhang et al. fabricated TCPP/UiO@SSM multifunctional membranes by in situ growing TCPP-doped UiO-66-NH_2_ (TCPP = tetrakis(4-carboxyphenyl)porphyrin) on stainless steel mesh (SSM), enabling gravity-driven oil-water/seawater separation and visible-light-driven bacterial inactivation [102]. Photosensitizers suffer a notable decline in reactive oxygen species production due to self-aggregation caused by strong π-π stacking—a critical bottleneck limiting their use in photocatalytic water purification and disinfection. As illustrated in Figure 11, Yun-Lan Li and co-workers designed and synthesized porous anthracene-based MOFs (GXNU-1, GXNU-2, GXNU-3) with excellent ROS-generating capacity, which can effectively inhibit the proliferation of Gram-negative and Gram-positive bacteria under light irradiation. Notably, masks and lab coats immersed in GXNU-1 aqueous solution for 15 s and exposed to 60 mW·cm^−2^ light for 4 min exhibited significant resistance to bacterial contamination [103].

In these non-medical applications, the environmental impact and degradation behavior of MOFs should also be considered. While their antimicrobial efficacy is beneficial, the release of metal ions or organic components into ecosystems requires careful evaluation to prevent potential ecological toxicity.

**Table 4:** Summary of Research Applications of MOFs in the Field of Antibacterial Science.

Application Category	MOF Material	Core Design/Strategy	Key Function & Outcome	Reference
5.1 Wound dressings and medical coatings	Cu-MOF (HKUST-1)	Electrostatic spinning of fibers with chitosan/PVA blends.	Excellent physicochemical properties, biocompatibility, and antimicrobial activity for full-layer skin repair.	[79]
	Ag@MOF	Dual-layer dressing: silver-loaded MOF/chitosan nanoparticles in the upper layer and PACS hydrogel in the lower layer.	Significantly accelerates wound healing, achieving more complete epithelialization and reducing inflammatory cells.	[79]
	Zn-MOF	Chitosan-based nanofiber scaffolds doped with tannic acid (TA).	Great potential in hemostatic wound care as a new antibacterial hemostatic wound dressing	[104]
	TA@ZIF-8 (TZ)	Complexed with oxidized bacterial cellulose (TBC) and MXene to form a hydrogel (TTZM) with a strong antimicrobial effect under 808 nm NIR irradiation.	Strong antimicrobial efficacy under 808 nm NIR irradiation for effective treatment of bacterial-infected wounds.	[80]
	Amino-functionalized nano-MOFs	MOFs were used as carriers and dynamic cross-linkers to form self-healing hydrogels with aldolylated alginates loaded with Cu NPs and curcumin.	Achieve synergistic anti-inflammatory and antimicrobial effects with good self-healing properties.	[81]
	CuBTC	Covalently immobilized on the surface of medical polypropylene (PP), releasing nitric oxide (NO).	Endowing surfaces with anti-fouling properties to inhibit bacterial adhesion on polypropylene.	[83]
	Cu-MOFs	Bionic coating with layer-by-layer self-assembly technology anchored to the inner surface of the PVC conduit.	Reduces non-specific adsorption of model proteins (anti-biofouling) and significantly inhibits platelet adhesion/activation (anti-platelet).	[84]
	Amino-functionalized Cu-MOF	Modification of thermoplastic polyurethane (TPU) by spin-coating and polyurethane prepolymer (PC) coating.	The release of NO demonstrates >96% antibacterial efficacy against Escherichia coli and Staphylococcus epidermidis.	[85]
	MOF-derived CuO@ZnO	Grafted on polydopamine (PDA) modified titanium alloy surface.	Controlled release of Zn^2+^/Cu^2+^ generates reactive oxygen species (ROS), effectively inhibiting bacterial biofilm formation and achieving a 99% kill rate against Staphylococcus aureus.	[86]
	Ag-MOF	Synthesized by a mild liquid phase method and integrated into acrylic coatings.	Continuous release of Ag^+^ achieves kill rates of 95.9% against *Escherichia coli* and 87.2% against *Staphylococcus aureus.*	[87]
5.2 Treatment of drug-resistant bacterial infections	UiO-66	Construction of the MXF@UiO-UBI-PEGTK nanosystem: loaded with antibiotic (moxifloxacin), targeted peptide (UBI), and ROS-responsive shell.	Possesses excellent biocompatibility, targeting capability, and synergistic bactericidal efficacy, specifically targeting biofilms and endophthalmitis.	[90]
	UiO-66-NH_2_	Forming UNMS nanocrystals with MoS_2_ via amidation yields positively charged particles.	Integrating photothermal, photodynamic, and peroxidase-mimetic activities, it generates reactive oxygen species under near-infrared light and exhibits broad-spectrum pH catalytic activity.	[91]
	Ga-MOF	Synthesized at room temperature, in situ loaded with the antimicrobial peptide (Melittin).	Achieves a synergistic antibacterial effect where the whole is greater than the sum of its parts, exhibits excellent biocompatibility, and accelerates the healing of MRSA-infected wounds.	[92]
	ZIF	The PLL modifies the ZIF via the ROS sensitive key, with internal loads CIP and CUR (ZIF/PLL-CIP/CUR).	Under blue light, ROS-responsive drug release generates ^1^O_2_/O_2_^−^, combining chemotherapy with photodynamic therapy to eliminate MRSA and biofilms.	[93]
	Zn-MOF	PDA-coated Zn-MOFs for enhancing the antibacterial efficacy of curcumin.	Small-sized MOFs effectively load and release curcumin, with PDA-Cur-Zn-MOFs inducing complete morphological distortion in bacteria.	[94]
	ZIF	Formation of the ZIF/PGA-C/M complex, featuring an enzyme-crosslinked polypeptide shell.	Effectively inhibits planktonic and biofilm MRSA, showing synergistic efficacy in a mouse skin infection model.	[88]
	ZIF-8	Constructing PMZMU nanoparticles: stem cell membrane encapsulation, incorporation of photosensitizer, acid-responsive ZIF-8 loading of polymyxin B.	Synergistic sonodynamic-nano-antimicrobial therapy under ultrasound targets Gram-negative drug-resistant bacteria, reduces inflammation, and improves survival rates.	[95]
	Multimetallic MOF	Integrating bacterial binding ligands (boric acid) and photosensitizers (porphyrins) into a single metal–organic framework.	Enhanced antimicrobial activity through synergistic action and tight physical gaps, effective elimination of drug-resistant bacteria.	[96]
	ZIF-67	Constructing an ONP@ZnO_2_@ZIF-67 (ONP@ZZ) core–shell structure.	As a “ROS factory”, it enables image-guided, in situ activation of photodynamic therapy (PDT) to eliminate drug-resistant bacteria.	[97]
5.3 Other antimicrobial applications	β-CD-MOF	Loaded with apigenin and corn protein (zein), a BCCZ composite membrane was fabricated.	Effectively inhibits Gram-positive and Gram-negative bacteria and fungi under high humidity conditions; Suitable for food packaging.	[98]
	MOFs	In situ formation of MOF-doped coatings on cellulose paper substrates.	Enhance hydrophobicity and provide long-lasting antibacterial effects; Active superhydrophobic paper-based packaging.	[89]
	Zn@MOF	As a carrier for volatile antibacterial essential oils (thymol).	An effective antimicrobial agent with potential for indirect application in the food sector.	[105]
	MOF-on-MOF (MN)	MOF-on-MOF heterojunctions prepared via simple ball milling.	For visible light-driven photoreduction of Cr(VI) and antimicrobial activity, treating wastewater containing Cr(VI) and bacteria.	[99]
	MIL-125(Ti)-NH_2_	Following modification, it is immobilized within the non-woven fabric via phase inversion to form a photocatalytic membrane.	Electrostatic capture of pathogens, photocatalytic inactivation, and maintaining long-lasting antibacterial efficacy even in darkness, for water purification.	[100]
	MIL-125(Ti)	Derivative synthesis of carbon-MIL-125 materials.	New perspectives for the design of efficient photocatalysts for water disinfection.	[101]
	TCPP/UiO-66-NH_2_	Multifunctional membranes fabricated by in situ growth on stainless steel mesh (SSM).	Achieving gravity-driven oil-water/seawater separation and visible light-driven bacterial inactivation.	[102]
	Anthracene-based metal–organic frameworks (GXNU-1/2/3)	Design and synthesis of porous anthracene-based metal–organic frameworks.	Possesses excellent ROS generation capacity, effectively inhibiting Gram-negative and Gram-positive bacteria, and can be used for treating items such as face masks and laboratory coats.	[103]

## 6. Conclusions and Future Prospects

Over the past decade, amid the dual drivers of the escalating traditional antibiotic resistance crisis and advancements in nanomaterial engineering, metal–organic frameworks have emerged as a pivotal breakthrough in antimicrobial materials. Endowed with tunable compositions, structural versatility, ultrahigh specific surface areas, and porosity [106], MOFs possess distinct advantages in combating drug-resistant bacteria and biofilms through a quadruple synergistic mechanism: sustained release of intrinsic metal ions (e.g., Zn^2+^/Cu^2+^), stimuli-responsive delivery of externally loaded antibiotics/enzymes, self-catalyzed reactive oxygen species (ROS) generation, and sterilization via light/electricity/heat energy conversion.

Although numerous in vitro and animal model studies have demonstrated the potent antimicrobial efficacy of MOFs, a significant gap remains between bench-scale promise and clinical applicability. Clinical validations of their efficacy in wound infections, osteomyelitis, and periodontitis have been reported: electrospun MOF/chitosan dressings accelerate diabetic ulcer healing by 200%; Mg-MOF orthopedic coatings simultaneously enhance osseointegration (with 27.8% higher bone density) and eliminate implant-associated infections; and ZIF-8 periodontal sustained-release systems achieve >95% biofilm clearance for drug-resistant strains [107]. Robust clinical trial data to validate their biocompatibility, long-term safety, and efficacy in human physiological environments are notably lacking.

Nonetheless, three core challenges remain in this field: bottlenecks in clinical translation (current research is largely confined to in vitro and animal models [108], lacking clinical trial evidence to validate biocompatibility and antimicrobial efficacy in human physiological environments); insufficient responsiveness to complex infections (most experiments target single bacterial species, with inadequate research on clinically prevalent multi-species mixed infections); and incomplete mechanistic understanding (e.g., ambiguous molecular dynamics of self-catalytic pathways) [109]. Moreover, the instability of certain MOFs under physiological conditions—such as hydrolysis, acid dissociation, or ion exchange—may lead to premature degradation, uncontrolled release of active components, and reduced antimicrobial durability, limiting their reliability in long-term or systemic applications. Additionally, while MOFs are often highlighted for their potential low toxicity, it is crucial to acknowledge that the sustained release of metal ions (e.g., Ag^+^, Cu^2+^, Co^2+^) may pose cytotoxicity risks at elevated concentrations and could lead to accumulation in tissues. Systematic evaluation of ion release kinetics, dose-dependent cytotoxicity, and long-term biodistribution is essential to ensure biosafety and facilitate clinical translation.

Future advancements should focus on three key directions: first, developing human-relevant infection models (such as 3D organoid co-culture systems) to simulate multi-species competitive microenvironments and verify the broad-spectrum antimicrobial efficacy of MOFs [110]; second, accelerating Phase I/II clinical trials to evaluate the long-term metabolic behavior and immune responses of MOFs in humans (e.g., PCN-224-mediated photodynamic therapy has completed safety pre-experiments in oral infection models) [111]; third, investigating and countering bacterial adaptive mechanisms—such as by integrating efflux pump inhibitors or ROS amplifiers into MOF structures—to preempt resistance development; finally, improving material stability through structural optimization (e.g., hydrophobic modification, robust metal-ligand pairing, or composite formation) to ensure controlled degradation and sustained antimicrobial action under physiological conditions. Furthermore, in situ characterization techniques should be employed (cryo-electron microscopy coupled with in situ Raman spectroscopy) to elucidate MOF–bacteria interface interactions, guiding the design of smart materials with linked multi-mechanisms, for instance, loading CRISPR-Cas9 gene-editing systems into ZIF-8 pores to simultaneously target and cleave resistance genes while activating photothermal sterilization, and achieving full-chain therapy encompassing “resistance blockade-precise sterilization-tissue repair.”

Through interdisciplinary integration (e.g., combining synthetic biology with microfluidic technology) and the establishment of clinical translation pipelines, MOF-based antimicrobial materials are poised to become a critical solution for combating antibiotic-resistant bacterial infections, driving a paradigm shift in antimicrobial therapy from “passive defense” to “active ecological regulation,” and ultimately alleviating global public health crises. At the same time, vigilance to the evolution of bacterial adaptations, fine-tuning of metal ion release profiles to minimize cytotoxicity, and continued innovation in the structural design of MOFs will be key to maintaining their efficacy and maximizing their value for practical applications.

## Figures and Tables

**Table 1:** Comparison of antimicrobial properties of metal–organic framework materials with other commonly used materials/nanomaterials.

Characteristic Dimension	Metal–Organic Frameworks (MOFs) [12,13,14,15,16,17,18,19,20,21,22]	Conventional Organic/Inorganic Compounds and Salts [1,2,3]	Single-Metal/Metal Oxide Nanoparticles (Such as nAg, nZnO, TiO_2_) [9,10,11]	Antibiotics [4,5,6,7,8]
Antimicrobial mechanism	Controlled release of metal ions; Generation of reactive oxygen species (ROS); Efficient loading and delivery of functional antimicrobial molecules;	Chemical action (e.g., redox); Disruption of cell membrane integrity.	Release metal ions (nAg, nZnO); Photocatalytically generate reactive oxygen species (e.g., TiO_2_);	Inhibits cell wall synthesis; Disrupts protein function; Inhibits nucleic acid replication, etc.
Structural adjustability/design flexibility	Pore size, shape, and functional groups can be precisely controlled	Fixed chemical structure	Limited control of size and shape	Dependent on the inherent chemical structure
Persistence of antimicrobial activity	Metal ions are slowly released through the framework degradation, providing a prolonged duration of action.	Prone to rapid depletion or deactivation	Inactivation due to rapid release or agglomeration of ions	Prone to degradation by drug-resistant enzymes, with a limited effective period.
Load and Synergy Capacity	High specific surface area/porosity, achieving synergistic enhancement	No load capacity	The surface may be minimally modified, but it has low load-bearing capacity and is prone to leakage.	No load required
Risk of bacterial resistance	Multi-Mechanism Synergy: Reducing the Risk of Single-Mechanism Induced Resistance	A single mechanism of action is prone to inducing drug resistance.	Dependent on ion release or photocatalysis, it may be risky for long-term use.	Misuse leads to a sharp rise in drug resistance.
Biocompatibility and Toxicity	Selection of biocompatible metals/ligands controls toxicity	Certain compounds exhibit relatively high toxicity.	High concentrations of metal ions may cause cytotoxicity and the risk of nanoparticle accumulation in the body.	Side effects such as allergic reactions, liver and kidney toxicity
Primary application areas	Antimicrobial Coatings & Implants; Targeted Drug Delivery Systems; Smart Wound Dressings; Food Active Packaging; Water Purification.	Surface disinfectant; Industrial preservative	Antibacterial Textiles; Antimicrobial Coatings; Personal Care Additives.	Clinical Infection Treatment; Livestock growth promotion.

**Table 2:** Advantages and disadvantages of various methods used for the production of metal–organic framework materials (MOFs).

Synthesis Method	Advantages	Disadvantages
Hydrothermal/Solvothermal [29,30,31,32]	The process is simple; MOFs with homogeneous morphology and high structural stability can be prepared, and the particles are well dispersed.	Requires high temperature and high pressure environments, limiting scalability for mass production.
Microwave-Assisted [33,34,35,36,37,39,48]	Highly efficient; fast reaction kinetics; Rapid and uniform heating can significantly reduce reaction time. High spatiotemporal yield (e.g., >1200 kg/m^3^/day); Good crystallinity and phase selectivity.	There exists a risk of localized overheating, which may adversely affect the nucleation and crystal growth of MOFs.
Electrochemical [37,38,40,41,42,43,44]	The release of metal ions/ligands can be precisely controlled. device integration is facilitated, and the crystalline quality of the prepared films is high	Constrained by large-scale production capacity
Mechanochemical [45,47]	Green and efficient; Fast reaction rate; easy to operate; high stability; suitable for industrialization; High temporal and spatial yields; continuous flow production is possible.	Challenges in improving material crystallinity.

**Table 3:** Advantages and disadvantages of different metal–organic frameworks (MOFs) compositions.

Type	Metal Center/Structural Features	Core Benefits	Main Limitations
Single-metal–organic frameworks [50,51,52,53,54]	Single metal ions (such as Ag^+^, Cu^2+^, Zn^2+^, Fe^3+^, Zr^4+^)	Simple structure, mature synthesis; various metal choices, can have both intrinsic antimicrobial properties; adjustable ligand function, easy to introduce specific functional groups.	Relatively monofunctional; stability may be inadequate; some metal ions are potentially cytotoxic.
Bimetallic metal–organic framework materials [56,57,58,59,60]	Synergistic interaction between two metal ions (e.g., Zn/Cu, Fe/Zr, Mg/Cu)	Significant synergistic effect, performance (e.g., catalytic activity) 1 + 1 > 2; strong designability of performance; easier integration of environmental response (e.g., photothermal, glucose response) and other smart functions.	Synthesis is more complex; synergistic mechanisms are poorly elucidated; biosafety still needs systematic assessment.
Multimetallic metal–organic framework materials [61,62,63,64,65,66,67,68,69,70,71]	Three or more metal ions (e.g., NiCoCu, PtPdRhAu)	Synergistic multifunctionality and extremely high performance in catalysis, antimicrobial, and other applications; a wide range of application areas (environment, energy, biomedicine).	Extremely challenging to synthesize; difficult to characterize and analyze; costly (especially with precious metal systems); lack of biosafety data.

## Data Availability

No new data were created or analyzed in this study. Data sharing is not applicable to this article.
